# Recent Progress in Doped g-C_3_N_4_ Photocatalyst for Solar Water Splitting: A Review

**DOI:** 10.3389/fchem.2022.955065

**Published:** 2022-07-13

**Authors:** Yilong Yang, Wantong Niu, Liyun Dang, Yanli Mao, Junshu Wu, Kaidong Xu

**Affiliations:** ^1^ School of Materials and Chemical Engineering, Henan University of Urban Construction, Pingdingshan, China; ^2^ Henan Province Key Laboratory of Water Pollution Control and Rehabilitation Technology, Henan University of Urban Construction, Pingdingshan, China; ^3^ Key Laboratory of Advanced Functional Materials, Ministry of Education, Faculty of Materials and Manufacturing, Beijing University of Technology, Beijing, China

**Keywords:** doping, G-C3N4, photoadsorption, band structure, water splitting

## Abstract

Graphitic carbon nitride (g-C_3_N_4_) photocatalysis for water splitting is harvested as a fascinating way for addressing the global energy crisis. At present, numerous research subjects have been achieved to design and develop g-C_3_N_4_ photocatalysis, and the photocatalytic system still suffers from low efficiency that is far from practical applications. Here, there is an inspiring review on the latest progress of the doping strategies to modify g-C_3_N_4_ for enhancing the efficiency of photocatalytic water splitting, including non-metal doping, metal doping, and molecular doping. Finally, the review concludes a summary and highlights some perspectives on the challenges and future research of g-C_3_N_4_ photocatalysts.

## Introduction

**FIGURE 1:**
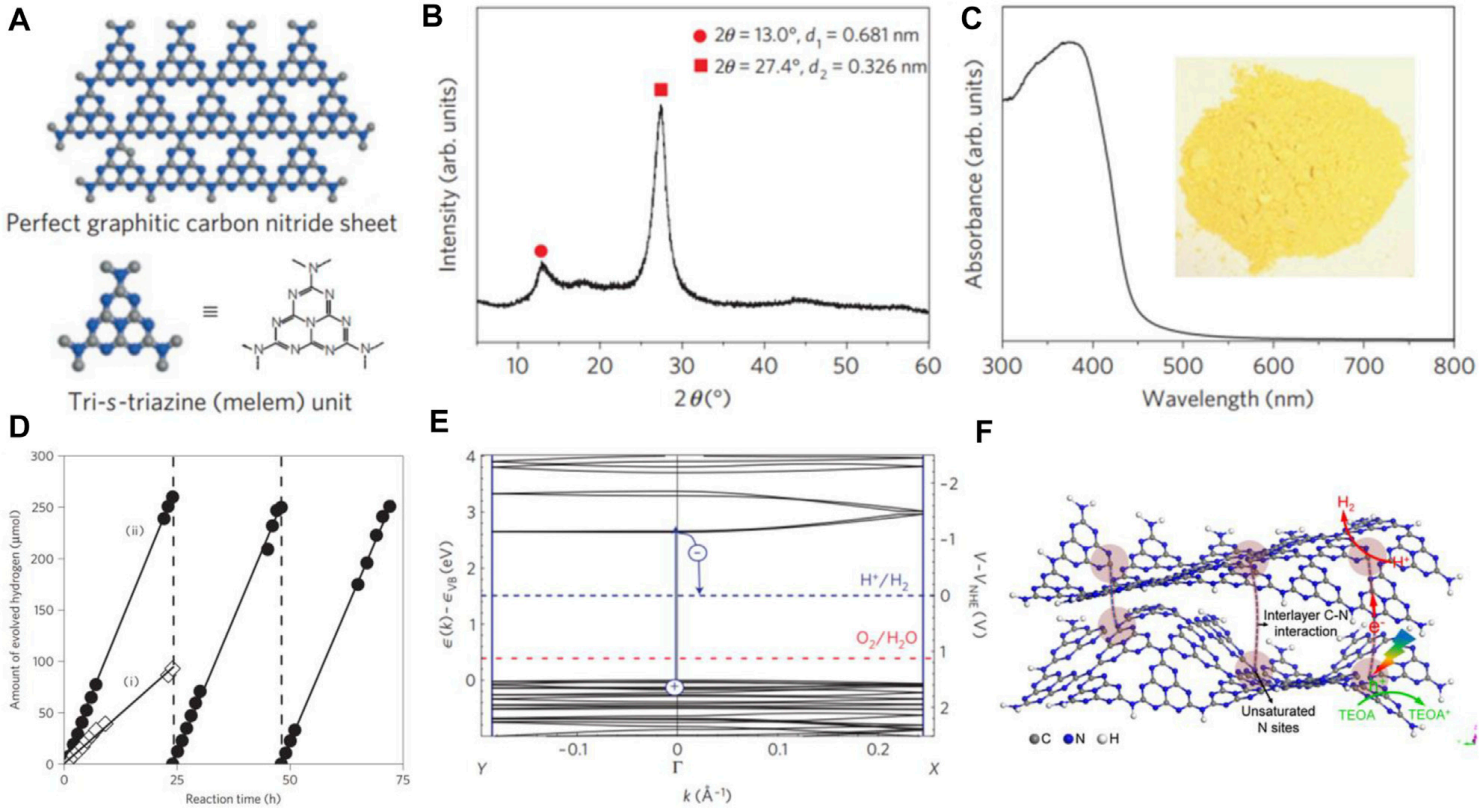
**(A)** Schematic diagram of a perfect g-C_3_N_4_ sheet constructed from melem units, **(B)** experimental XRD pattern of g-C_3_N_4_, **(C)** ultraviolet–visible diffuse reflectance spectrum of the g-C_3_N_4_. Inset: Photograph of g-C_3_N_4_, **(D)** typical time course of H_2_ production from water containing 10 vol% triethanolamine as an electron donor under visible light (of wavelength longer than 420 nm) by (i) unmodified g-C_3_N_4_ and (ii) 3.0 wt% Pt-deposited g-C_3_N_4_ photocatalyst, **(E)** density-functional-theory band structure for polymeric melon calculated along with the chain (*Γ–X* direction) and perpendicular to the chain (Y–*Γ* direction). The position of the reduction level for H^+^ to H_2_ is indicated by the dashed blue line, and the oxidation potential of H_2_O to O_2_ is indicated by the red dashed line just above the valence band. Copyright 2009, Springer Nature; **(F)** illustration of the charge transfer in g-C_3_N_4_ nanosheets under visible light. Copyright 2017, Elsevier.

The global energy demands and environmental crisis have stimulated tremendous research on the exploration of green and renewable energy due to awareness of energy conservation and environmental protection (Miao et al., 2022). Since titanium dioxide (TiO_2_) was discovered as the photoanode for photoelectrochemical (PEC) water splitting(Fujishima and Honda, 1972), semiconductor-based photocatalysis for solar hydrogen production has seen an upsurge in global interests (Wang and Wang, 2019; Zada et al., 2020; Qi et al., 2021; Wei et al., 2021). However, it is still very challenging to achieve high solar-to-hydrogen (STH) conversion efficiency toward practical applications. To make high utilization of solar energy, the exploration of visible-light-active photocatalysts is highly desirable. In 2009, Wang et al. developed the pioneering work on g-C_3_N_4_ for visible-light–driven photocatalytic water splitting(Wang et al., 2009), and g-C_3_N_4_-based photocatalysis has drawn considerable attention in the last decade (Xiao et al., 2021; Xing et al., 2021; Chen et al., 2022a) (Figures 1A–E). For unification in this study, we will consider the two kinds of materials with triazine (C_3_N_3_) unit or tri-s-triazine (C_6_N_7_) unit (Figure 1A) to name as g-C_3_N_4_. g-C_3_N_4_ affords a lamellar structure consisting of C and N atoms which is similar to graphene and can be traced back to the original form of “melon” found by Berzelius and Liebig in 1834 (Liebig, 1834). Unlike TiO_2_, g-C_3_N_4_ affords a narrow bandgap of 2.7 eV (Figure 1C) with the valance band (VB) position at +1.6 eV and conduction band (CB) position at −1.1 eV *vs*. normal hydrogen electrode (NHE) (Wang et al., 2009) (Figure 1E). This enables g-C_3_N_4_ to drive photocatalytic reaction using visible light.

Generally speaking, the g-C_3_N_4_ photocatalyst possesses all the following abilities: stability, non-toxicity, abundant source, visible-light-responsive absorption, and easy to control and modify. As a fantastic visible-light–driven photocatalyst, its remarkable property has been conducted on widely photocatalytic applications, such as H_2_ evolution from water (Zhao et al., 2017a; Yu et al., 2021; Zhao et al., 2022), O_2_ evolution (Yang et al., 2019; Zhao et al., 2019; Xu et al., 2021), overall water splitting (Chen et al., 2019; Bhagat and Dashora, 2021; Wu et al., 2021), photodegradation of pollutants (Xiong et al., 2016; Duan et al., 2019; Jing et al., 2021), CO_2_ reduction (Jiang et al., 2018; Yang et al., 2020a; Cheng et al., 2020), organic synthesis (Devthade et al., 2018; Camussi et al., 2019; Lima et al., 2019), and photoelectrocatalysis (Karimi-Nazarabad et al., 2019; Wu et al., 2020; Huo et al., 2021). However, the three crucial issues of light absorption efficiency, charge separation and transfer efficiency, and surface reaction efficiency still restrict the development of high-performance g-C_3_N_4_ photocatalysis, which is fairly difficult to achieve by pristine g-C_3_N_4_.

Although the g-C_3_N_4_-based photocatalysis has been fully discussed in many recent review articles(Li et al., 2020a; Li et al., 2021a; Chen et al., 2021; Xing et al., 2021; Zhu et al., 2021; Chen et al., 2022b), a relative focus review about doping strategies to modify g-C_3_N_4_ for improving the efficiency of water splitting is still lacking. Compared with other effective strategies for modification of g-C_3_N_4_, like heterojunction construction, defect introduction, and nanostructure controlling, the modification process of g-C_3_N_4_ can be comparatively simplified by element doping to tune the band gap, which considerably broadens the light absorption and accelerates the electron-hole pair separation (Li et al., 2020b). This work overviews the recent advances of g-C_3_N_4_ materials focusing on efficient photocatalytic water splitting in doping strategies for modifying carbon nitride including non-metal doping, metal doping, and molecular doping. This review also aims to present a general summarization in boosting the g-C_3_N_4_ photocatalyst to seek new inspiration for material science.

### Basic Properties of Carbon Nitride for Solar Water Splitting

Under irradiation, the electrons of the g-C_3_N_4_ photocatalyst can be excited from the VB to CB by absorbing the photons with the energy (*hν*) higher than the bandgap energy, wherein holes are left in the VB (Luo et al., 2016). The large parts of the photoexcited charge carriers will combine rapidly, and only a small part of photogenerated electrons and holes can be transferred to the surface of g-C_3_N_4_ to involve the reaction. Then, the water molecules can be reduced with the photoexcited electrons for H_2_ evolution and oxidized by the photoexcited holes for O_2_ generation during the photocatalytic reaction. For H_2_ evolution, the CB potential of the photocatalyst should be more negative than the H_2_ reduction potential, while the VB potential should be more positive than the water oxidation potential for the O_2_ evolution from water. The g-C_3_N_4_ photocatalyst possesses a CB of −1.1 eV and a VB of 1.6 eV is fit for splitting water to H_2_ and O_2_.

So far, visible-light photoadsorption, high chemical stability, appropriate CB and VB potentials, and strong photocatalytic activity make g-C_3_N_4_ be the most widely focused in photocatalytic water splitting. Nevertheless, the efficiency of water splitting by g-C_3_N_4_ is still low, mainly due to limited photoadsorption in the visible-light region, limited ability of electron transport along or across the g-C_3_N_4_ sheets (Figure 1F), and recombination of photoexcited electron-hole pairs.

In a word, due to dramatic development of g-C_3_N_4_ photocatalysts in the STH conversion field, a review focusing on the photocatalytic water splitting is still necessary to provide researchers a state-of-art progress in this dynamic research field. This review presents a brief discussion of the current doping research, accompanied with the challenges and future direction of g-C_3_N_4_ photocatalysts for photocatalytic applications.

### Doping Strategies for Modifying Carbon Nitride

To develop advanced g-C_3_N_4_ photocatalysts, a doping strategy is considered an appealing way to modulate physicochemical properties such as band structure tailoring and light adsorption improving, which, therefore, enhance the performance of photocatalyst (Patnaik et al., 2021). Based on the arrangements of doping elements, the classification of doping can be divided into non-metal doping, metal doping, and molecular doping.

### Non-Metal Doping

The fabrication of non-metal doped carbon nitride is effective in modulating the electronic structure of g-C_3_N_4_ by distorting the π-conjugated orbital. Boron-doped g-C_3_N_4_ was prepared by microwave heating for hydrogen evolution, and boric acid was used as a doping source combined with melamine and urea for thermal condensation (Chen et al., 2018). g-C_3_N_4_ nanosheets can be *in situ* modified by boron atoms to improve the photoadsorption, hinder the annihilation of charge carriers, and prolong the lifetime of photogenerated electrons. Combining g-C_3_N_4_ with strongly electronegative dopants such as fluorine to form an F-doped material not only raised the valence band but also affected the thermodynamic driving force for H_2_ reduction (Zhu et al., 2017). Fluorinated (F) carbon nitride solids were also reported with excellent visible-light photocatalytic activity (Shevlin and Guo, 2016). Fluorination not only provided a modified texture but also enabled effective adjustment of the electronic band structure, which was demonstrated by improved activities. To further explore the structural distortion-dependent photoreactivity, it is more desirable to exploit co-doping that may be a good attempt to further improve the photocatalytic activity of g-C_3_N_4_ through the synergistic effects of the two dopants. For instance, B/F co-doped g-C_3_N_4_ was fabricated by polymerizing urea and ionic liquids ([Bmim][BF_4_]), which was used as the texture modifier and dopant source (Lin and Wang, 2014). This research leads in a new one-pot fabrication of B and F co-doped g-C_3_N_4_.

In addition to F, other halogen elements we cannot ignore are chloride (Cl), bromine (Br), and iodine (I) in the doping area. Br-doped g-C_3_N_4_ was also successfully synthesized by using ionic liquid as the Br source and soft-template for photoredox water splitting (Zhao et al., 2017b). The Br-doping tuned light absorption and band structure without destroying the major construction of the g-C_3_N_4_ polymer. Similarly, I doped g-C_3_N_4_ materials also lead to positive effects that enlarged the specific surface area, enhanced optical absorption, narrowed the bandgap, and accelerated the photoinduced charge carrier transfer rate, leading to an increasing H_2_ evolution rate (Iqbal et al., 2020).

**FIGURE 2:**
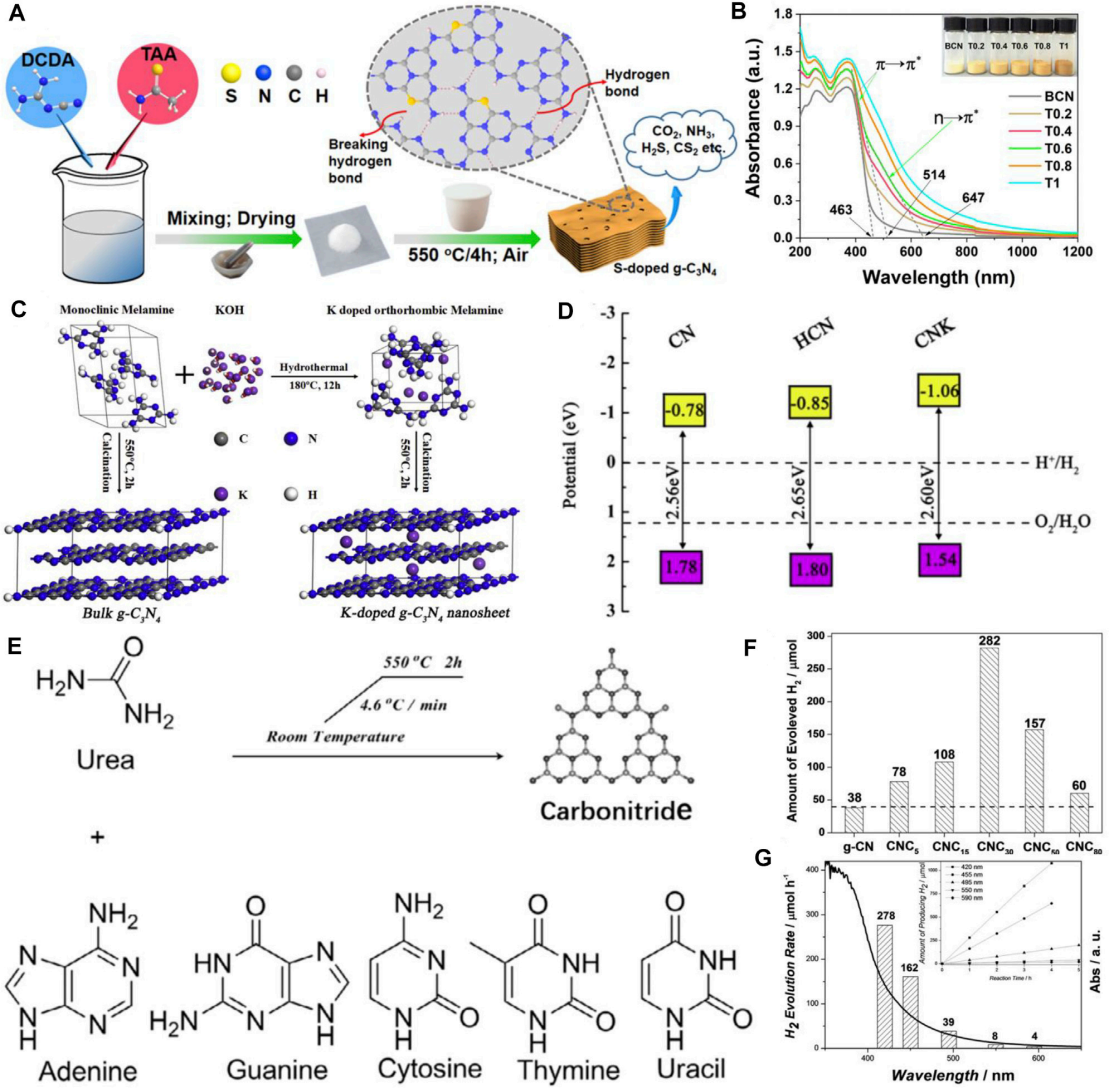
**(A)** Schematic illustration for the formation of multi-layered cake-like porous g-C_3_N_4_ with broken hydrogen bonds by S-doping, **(B)** UV–visible diffuse reflectance spectra of samples. Copyright 2020, Elsevier; **(C)** Schematic illustration of the KOH-assisted hydrothermal-reformed melamine strategy for achieving simultaneous K-doping and exfoliation of g-C_3_N_4_, **(D)** band structure alignments for the CN, HCN, and CNK sample. Copyright 2018, Elsevier; **(E)** Chemical production of polymeric carbon nitride semiconductors from nucleobases and urea, **(F)** Effect of cytosine amount on the HER, **(G)** wavelength dependence of the HER with CNC_30_ loaded with 3 wt% Pt. Inset: time-dependence of the HER with CNC_30_ at different irradiation wavelengths. Copyright 2017 John Wiley and Sons, Inc.

In addition, introducing other non-metal elements are also effective strategies in promoting the photocatalytic property (Deng et al., 2019; Li et al., 2021b; Li et al., 2021c; Liu et al., 2021; Yan et al., 2022). For instance, carbon (C) self-doped g-C_3_N_4_ was prepared *via* a combined method of melamine-cyanuric acid complex supramolecular pre-assembly and solvothermal pre-treatment (Li et al., 2021b). The H_2_ evolution rate for optimized g-C_3_N_4_ was 18 times higher than that of bulk g-C_3_N_4_, and the enhanced performance derives from the extended optical absorption, accelerated photoactivated charge carrier separation, and transfer efficiency. Unique oxygen-doped g-C_3_N_4_ materials were synthesized, which realized the synergetic control of the electronic structure and morphology and possessed the advantages of enlarged surface area, increased exposed active edges, and improved separation efficiency (Yan et al., 2022). Some other high-performance non-metal doped materials including phosphorus-doped g-C_3_N_4_(Yang et al., 2018; Lin et al., 2020; Zhao et al., 2020) and sulfur-doped g-C_3_N_4_ (Li et al., 2019a; Yang et al., 2020b; Long et al., 2020) have also been developed. For instance, Yang et al. reported a flower-like P-doped g-C_3_N_4_ which was prepared using phosphoric acid and cyanuric acid-melamine complex, which served as the P source and the precursor of g-C_3_N_4_, respectively, (Yang et al., 2018). The as-prepared P-doped g-C_3_N_4_ showed a high visible-light photocatalytic H_2_ evolution rate of 256.4 μmol h^−1^ 50 mg^−1^ and almost 24-folds higher than those of the pristine g-C_3_N_4_. A sulfur (S) doped-g-C_3_N_4_ nanosheet with terminal-methylate was presented with a tunable bandgap (Li et al., 2019a). The VB near the Fermi level was split due to S atoms into methylated melon units, which generated a new empty mid-gap electronic state and improved the light-responsive property up to 700 nm. Furthermore, the photocatalytic activity restricted by intralayered hydrogen bonds should also be considered. Yang et al. reported an S-doped g-C_3_N_4_ through poly-condensation and the mixture of dicyandiamide and thioacetamide, resulting in greatly enhanced visible-light-response ability and n → π* electron transition. The substituting of sp2-hybridized N with S atoms contributed to break intralayered hydrogen bonds, which resulted in photocatalytic H_2_ production (Yang et al., 2020b) (Figures 2A,B).

### Metal Doping

Due to abundant orbital electrons, the electron structure and optical property of g-C_3_N_4_ can also be affected by metal element doping, leading to focusing on the metal doping method. The cobalt (Co)-doped g-C_3_N_4_ nanosheet was investigated to provide more separation centers by forming Co–N bond, which can celebrate charge transfer and enhance photocatalytic performance (Yang et al., 2021). Moreover, potassium-modified g-C_3_N_4_ (K-g-C_3_N_4_) nanosheets were synthesized (Sun et al., 2019) (Figures 2C,D). Photocatalytic H_2_ production tests under visible light irradiation showed high photocatalytic activities of K-g-C_3_N_4_ nanosheets (up to about 13-folds higher than that of original g-C_3_N_4_) as well as an apparent quantum efficiency (AQE) of 6.98% at 420 nm. In addition, lanthanum (La) and Co co-doped g-C_3_N_4_ was prepared by the wet impregnation method (Tasleem and Tahir, 2021). The H_2_ evolution by La/Co co-doped-g-C_3_N_4_ showed the highest H_2_ production of 250 μmol g^−1^h^−1^ among the samples, which was 2.5, 1.35, and 1.25 times higher than that of original g-C_3_N_4_, La-g-C_3_N_4,_ and Co-g-C_3_N_4,_ respectively. The enhanced activity can be contributed to the celebrated charge separation, which was originally the electron trapping capability of La and Co.

### Molecular Doping

Heteroatom doping as discussed above is often used to modulate the atomic and band structure of g-C_3_N_4_ to promote light harvesting and celebrate electron-hole separation and transfer. Especially, integrating another structure-matching aromatic structure with g-C_3_N_4_ is a unique method to tune the intrinsic features (Yang et al., 2017; Li et al., 2018; Li and Zhang, 2018; Li et al., 2019b; Li et al., 2019c; Liu et al., 2019; Li et al., 2020c; Jiang et al., 2021; Zhou et al., 2021). The thermal co-polymerization of the aromatic comonomers and precursors of g-C_3_N_4_ can narrow the band gaps of g-C_3_N_4_, which extends the visible light absorption edge to enhance the utilization of sunlight. For instance, Liu et al. designed in-plane benzene-ring doped g-C_3_N_4_ nanosheets by copolymerizing urea and 4, 4′-sulfonyldiphenol. It exhibited dramatic H_2_ generation efficiency with a PHE rate of 12.3 mmol h^−1^ g^−1^, which was almost 12-folds higher than that of intrinsic g-C_3_N_4_ and the AQE of 17.7% at 420 nm (Jiang et al., 2021). Moreover, copolymerization of urea and naphthoic acid has been conducted to construct an aromatic ring–doped g-C_3_N_4_, which was an effective strategy to extend the *π*-conjugated system for visible light absorption and elevate the efficiency of charge transfer (Li et al., 2020c). In addition, Yang et al. enriched the construction of g-C_3_N_4_ by using nucleobases (adenine, guanine, cytosine, thymine, and uracil) and urea to energize the production of the charge carrier with light irradiation, which inducted photoredox reactions for stable H_2_ evolution (Zhou et al., 2021) (Figures 2E–G).

## Conclusion and Outlook

This review presents a promising visible-light–driven photocatalyst, g-C_3_N_4_, benefiting from its unique microstructure, resistance against acids, and bases and fantastic band structure. Nevertheless, the pristine g-C_3_N_4_ suffers from some shortcomings, including limited photoadsorption capability and fast recombination of photoexcited electron-hole pairs, which largely restricts practical applications. The present review depicts a focus review on the doping strategies to design efficient g-C_3_N_4_ in the use of photocatalytic water splitting. In summary, doping can introduce the impurity levels in the band gap region to create a new band edge potential, which can extend the spectral response region with decreased band gap. In addition, the hetero dopants get settled either in the lattice or insert in the interlayers of g-C_3_N_4_, inducing the formation of hybridized orbitals. The hybridization between g-C_3_N_4_ and dopant orbital remarkably affects the charge transportation, life time of charge carriers, and the photocatalytic performance of g-C_3_N_4_. In short, doping is a feasible and effective strategy to regulate photo-absorbance, redox potentials, and transfer of photo-induced charge carriers and one of the attractive strategies to tune the physicochemical properties of g-C_3_N_4_.

To date, doping provides an innovative approach to promote the efficiency of g-C_3_N_4_ photocatalyst. However, some issues like nonuniform doping, formation of surface trapping center, or low oxidizing and reducing capability resulting from narrowing the bandgap still existed, while the mechanisms in this field are at the primary stage and further systematic investigations are still needed because of the relatively low visible-light photocatalytic efficiency, which is far from the requirements of practical applications. Some issues that must be resolved for doped g-C_3_N_4_ photocatalyst involve the fact that 1) the doping mechanisms of enhanced photocatalytic property is not clear. For example, many explanations of doping technology still stay at the stage of “the enhanced photocatalytic activity is contributed to the doping method” with no discussion about mechanism and essential meaning of element doping. 2) It is still challenging to bring forth new ideas on doping methods, and finding the right balance of lower redox ability and higher photocatalytic activity is highly desired. To overcome the challenges, lots of attempts are still needed. The heteroatom-doping assisted with theoretical simulation calculation can be a feasible method to analyze the doping effect. Especially, it is significant to develop a crystalline g-C_3_N_4_ (CCN) doped by metals or non-metals, which improves the charge separation, increases the reactive facet exposing, and shows dramatic photocatalytic water splitting performance. Furthermore, a broad range of heterostructures, including quantum dots/g-C_3_N_4_ junction, polymer/g-C_3_N_4_ junction, semiconductor/g-C_3_N_4_ junction, cocatalyst modification of single atoms and defects engineering, as well as nanostructure and crystalline control, should also be considered for improved photocatalysis to increase the photoabsorption, accelerating the charge separation and transfer, elongating the charge carrier lifetime, and boosting the photocatalytic water splitting. Focusing on the perspective of green and renew energy, it is no doubt that g-C_3_N_4_-based photocatalyst will draw more attention on the research of water splitting in the future.
